# IL-35 is critical in suppressing superantigenic *Staphylococcus aureus*-driven inflammatory Th17 responses in human nasopharynx-associated lymphoid tissue

**DOI:** 10.1038/s41385-019-0246-1

**Published:** 2020-01-02

**Authors:** Rong Xu, Rebecca K. Shears, Ravi Sharma, Madhan Krishna, Christopher Webb, Richard Ali, Xiaoqing Wei, Aras Kadioglu, Qibo Zhang

**Affiliations:** 1 Department of Clinical Infection, Microbiology and Immunology, Institute of Infection and Global Health, University of Liverpool, Liverpool, UK; 2 ENT Department, Alder Hey Children’s Hospital, Liverpool, UK; 3 ENT Department, Royal Liverpool and Broadgreen University Hospitals, Liverpool, UK; 4 Institute of Tissue Engineering and Repair, School of Dentistry, College of Biomedical and Life Sciences, Cardiff University, Cardiff, UK

## Abstract

The human nasopharynx is frequently exposed to microbial pathogens, including superantigen-producing *Staphylococcus aureus* (SAg-*Sau*), which activates potent pro-inflammatory T cell responses. However, cellular mechanisms that control SAg-*Sau*-driven T cell activation are poorly understood. Using human nasopharynx-associated lymphoid tissue (NALT), we show that SAg-*Sau* drove a strong Th17 activation, which was associated with an impaired CD4^+^ T cell-mediated immune regulation. This impairment of immune control correlated with a significant downregulation of interleukin-35 (IL-35) expression in tonsillar CD4^+^ T cells by SAg-*Sau*. Supplementing recombinant IL-35 suppressed SAg-*Sau*-activated Th17 responses, and this IL-35-mediated suppression positively correlated with the level of Th17 activation. Interestingly, SAg-*Sau* stimulation induced Foxp3^+^ Treg expansion and interleukin-10 (IL-10) production, which effectively suppressed the Th1 response, but failed to control the activation of Th17 cells. Overall, our results reveal an aberrant T cell regulation on SAg-*Sau*-driven Th17 activation and identify IL-35 as a critical cytokine to control superantigenic *S.aureus*-activated Th17 responses.

## Introduction

*Staphylococcus aureus* (*Sau*) is a commonly identified bacterial colonizer of the nasopharyngeal mucosa in humans.^1^ Up to half of the *Sau* isolates are known to be superantigenic, and produce various superantigens, including toxic shock syndrome toxin (TSST) and staphylococcal enterotoxins (SEA, SEB, SEC, SED, and SEE).^2,3^ These superantigens are extremely potent in stimulating poly-clonal T cell activation via cross-linking the MHC Class II of antigen-presenting cells (APC) and T cell receptor Vβ chain, which is not restricted to antigen specificity.^4^

Nasopharyngeal carriage of *Sau* increases the risk of invasive infections such as pneumonia, endocarditis and bacteraemia.^5^ SAg-*Sau* infection could cause toxic shock through the release of superantigens which elicit potent T cell activation and a “cytokine storm”.^6^ Further, SAg-*Sau* colonization has been associated with a range of inflammatory/autoimmune conditions, including asthma, chronic rhinosinusitis, Wegener’s granulomatosis (WG) and multiple sclerosis (MS).^3,7–9^

Nasopharynx-associated lymphoid tissues (NALT) are mucosal immune organs in the upper respiratory tract and are known induction sites for immunity against a number of respiratory pathogens. The exposure to a large number of microbial antigens results in a substantial number of proinflammatory T cells in NALT which could potentially lead to a highly inflammatory response in the presence of SAg-*Sau.*^10^ It is therefore important to understand how SAg-*Sau*-driven inflammation is controlled in the distinct immune environment of NALT, to better inform new strategies in the management of SAg-*Sau* associated inflammatory diseases.

Staphylococcal superantigens mainly trigger Th1 and Th17 responses characterized by massive production of pro-inflammatory cytokines, such as IFNγ, IL-17A, and TNF-α.^11^ IFNγ-producing Th1 cells were initially thought to play a central role in inflammatory/autoimmune diseases.^12^ However, subsequent findings showed genetic depletion of IFNγ in murine models of experimental autoimmune encephalomyelitis (EAE) enhanced disease severity and that would argue against this hypothesis.^13^ Accumulating evidences support a more central role for Th17 cells in mediating inflammatory/autoimmune diseases.^14^ By inducing neutrophil influx and enhancing production of a wide spectrum of inflammatory cytokines and chemokines, activation of Th17 cells promotes clearance of microbes, but also causes inflammation-driven tissue damage.^14,15^ Nasal carriage of SAg-*Sau* has been linked to WG, MS and rheumatoid arthritis (RA), and Th17 cells are known to play a critical role in the development of those diseases.^3,9,16–18^ Tight regulation of Th17 activation is needed to control the development of inflammatory/autoimmune diseases associated with SAg-*Sau* infection.

Foxp3^+^CD25^+^Tregs are the major CD4^+^ T cell population regulating over-activated inflammatory responses and maintaining immune tolerance.^19^ Staphylococcal superantigens have been shown to expand Foxp3^+^ Tregs in human PBMCs.^20,21^ However, whether SAg-*Sau*-activated proinflammatory T cell responses are controlled by Tregs is debated.^21,22^ IL-10 is a well-established immune suppressive cytokine produced by a number of immune cells including Foxp3^+^ Tregs and Foxp3^−^ type 1 regulatory cells (Tr1).^23^ A recent study demonstrates that mice infected with *Sau* exhibit enhanced IL-10 production which in turn inhibits the Th17 differentiation and therefore permits *Sau* systemic reinfection.^24^ While IL-10 is able to inhibit Th17 differentiation induced by *Sau*, the role of IL-10 in regulating *Sau*-activated memory Th17 responses is unclear.

IL-35 is a member of the IL-12 cytokine family, which is made of two shared subunits, IL-12A and EBI3.^25^ Unlike most of the other pro-inflammatory cytokines in the IL-12 family, IL-35 has been shown to suppress Th17 cell activation in animal models of autoimmune/inflammatory disorders.^25–27^ Inducible IL-35-producing regulatory T cells (iTr35s) have been identified, showing potent regulatory control over effector T cells.^26^ To date, whether IL-35 plays a role in *Sau*-activated pro-inflammatory T cell responses has not been reported.

Here, we show SAg-*Sau*-activated Th17 responses in human NALT were not suppressed by Foxp3^+^ Tregs and IL-10. SAg-*Sau* stimulation significantly downregulated IL-35 expression in the tonsillar CD4^+^ T cells, and exogenous IL-35 suppressed highly activated Th17 responses elicited by SAg-*Sau*. Our results support a critical role of IL-35 in suppressing SAg-*Sau*-driven inflammatory Th17 responses.

## Results

### SAg*-Sau* activates a potent Th17 response in human tonsillar MNCs

**Fig. 1:**
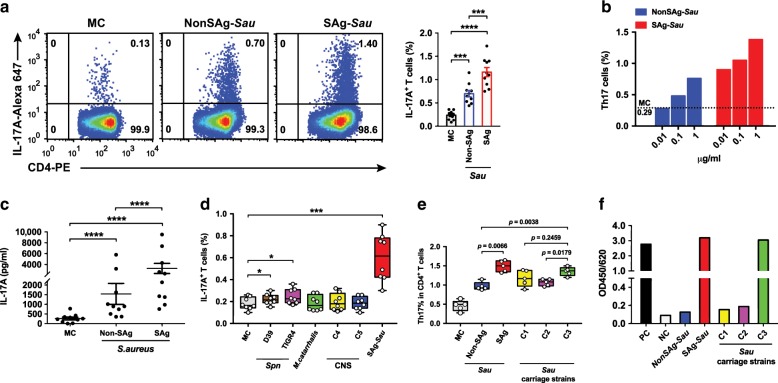
SAg-*Sau* activates a potent Th17 response in human tonsillar MNCs. **a**, **b**, **d**, **e** Intracellular cytokine analysis of IL-17A-expressing CD4^+^ T cells (Th17) in isolated human tonsillar MNCs 48 h following bacterial CCS (1 µg/ml) stimulation, compared to media control (MC) MNCs. **a** Dot plots were gated on CD4^+^ T cells and numbers in the top right quadrants indicate the percentage of Th17 cells within the CD4^+^ T cell population. Data were analyzed using paired *t*-test and displayed in mean ± SEM, *n* = 10. **b** Dose-dependent Th17 responses activated by NonSAg-*Sau* and SAg-*Sau,* respectively. Results are representative of 3 individual samples. **c** IL-17A concentration in tonsillar MNCs culture supernatants were measured by ELISA and samples assayed in duplicates. Data displayed is individual data points with mean ± SEM, *n* = 10. Paired *t*-test was performed on log-transformed data. **d** Box and Whiskers plot showing the percentage of Th17 cells within CD4^+^ T cells in tonsillar MNCs stimulated with *Spn, M. catarrhalis*, coagulase-negative staphylococcus (CNS, C4 and C5) and SAg-*Sau,* respectively. **e** The percentage of Th17 cells within CD4^+^ T cell population was summarized for tonsillar MNCs activated by NonSAg-*Sau*, SAg-*Sau,* and *Sau* carriage strains (C1, C2, and C3). Data (**d, e**) was displayed in median (center line), upper and lower quartiles (box limits) and minimum to maximum range (whiskers). 8 (**d**) and 5 (**e**) individual samples were tested and analyzed. **f** Staphylococcal enterotoxin A-E level in *Sau* strains (PC, positive control. NC, negative control), test was performed in duplicate. *p *<* 0.05, **p < 0.01, ***p < 0.001, ****p < 0.0001.

To examine whether SAg-*Sau* activates Th17 responses in human NALT, tonsillar mononuclear cells (MNCs) were stimulated with bacterial culture supernatant of *Sau*. A potent Th17 response was detected in tonsillar MNCs following stimulation with SAg-*Sau* (Fig. 1a). The Non-Superantigenic *Sau* (NonSAg-*Sau*) strain also activated a Th17 response, although significantly less than SAg-*Sau* stimulation (Fig. 1a). A dose-dependent Th17 response was shown following both NonSAg-*Sau* and SAg-*Sau* stimulation (Fig. 1b). Increased IL-17A production in the cell culture supernatant following stimulation was confirmed by ELISA (Fig. 1c). We then compared the Th17 responses activated by SAg-*Sau* with other frequently identified bacterial colonizers in the nasopharynx. *Streptococcus pneumoniae* (*Spn*) elicited a weak Th17 response, and no significant Th17 response was activated by *M. catarrhalis* and coagulase-negative staphylococcal strains (Fig. 1d). To further examine whether SAg-*Sau* carriage isolates from the nasopharynx also activated strong Th17 responses, total enterotoxin A-E level in the bacterial culture supernatant from *Sau* carriage isolates C1, C2 and C3 were measured by ELISA, and Th17 responses activated by these carriage strains were examined. C3 strain, which contained a similar level of enterotoxins as SAg-*Sau*, also activated a strong Th17 response, higher than NonSAg-*Sau*. C1 and C2 produced low level of enterotoxin A-E and activated Th17 response to a similar extent as NonSAg-*Sau* (Fig. 1e, f). Compared to C3, both C1 and C2 appeared to activate a lower Th17 response although it did not reach significance for C1 (Fig. 1e). Our data suggest *Sau*, particularly superantigenic strains, can activate potent Th17 responses in human NALT.

### SAg-*Sau*-activated Th17 responses are not suppressed by Foxp3^+^ Tregs

**Fig. 2:**
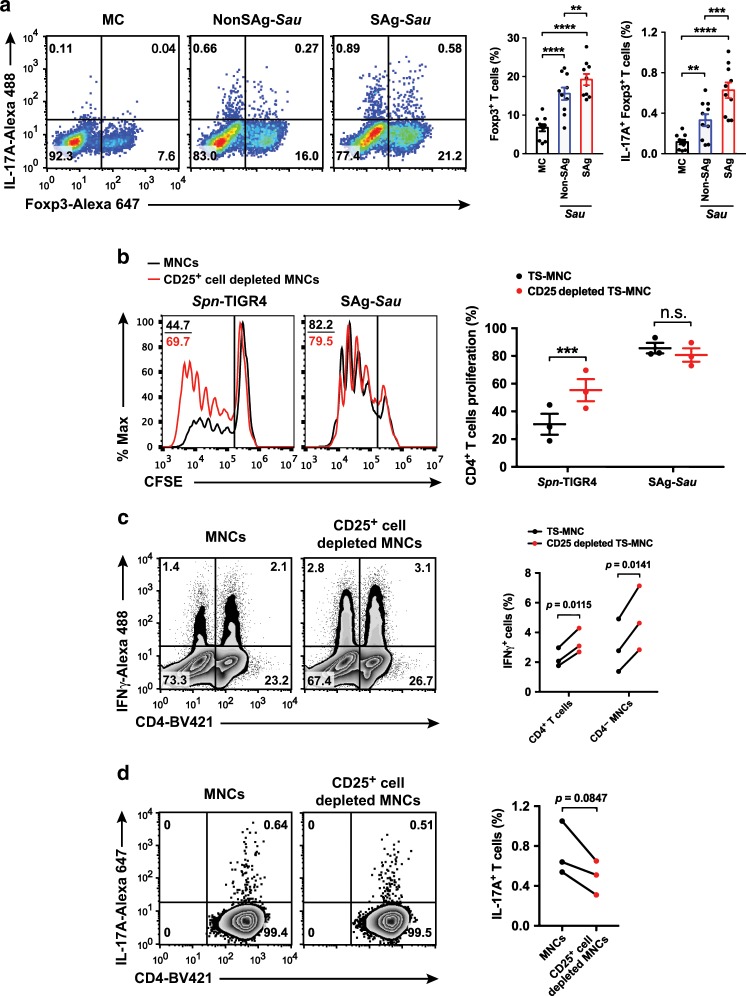
SAg-*Sau*-activated Th17 responses are not suppressed by Foxp3^+^ Tregs. **a** Analysis of Treg expansion and IL-17A-expressing Tregs in isolated human tonsillar MNCs 48 h following bacterial CCS (1 µg/ml) stimulation. Dot plots were gated on CD4^+^ T cells and numbers in bottom right and top right quadrants indicate the percentage of IL-17A^−^ Foxp3^+^ and IL-17A^+^ Foxp3^+^ cells, respectively, within the CD4^+^ T cell population. Data is displayed as mean ± SEM by column bar graphs and analyzed using paired *t*-test, *n* = 10. **b** Unfractionated MNCs and CD25^+^ cell depleted MNCs were labeled with CFSE in order to quantify CD4^+^ T cell proliferation 5 days after SAg-*Sau* CCS (1 µg/ml) stimulation. Histogram plots were gated on CD4^+^ T cells showing the median fluorescent intensity (MFI) of CFSE and numbers in the top left corners indicate percentage of proliferated CD4^+^ T cells. Data points with mean ± SEM are shown in the dot plot on the right with 3 individual samples tested. **c, d** IL-17A and IFNγ expression after 48 h of SAg-*Sau* CCS (1 µg/ml) stimulation for unfractionated MNCs and CD25^+^ cell depleted MNCs. Results were summarized from 3 individual samples. **c** Zebra plots were gated on lymphocytes and numbers in top left and right quadrants indicate the percentage of IFNγ^+^ CD4^−^ lymphocytes and IFNγ^+^ CD4^+^ T cells (Th1) in total lymphocytes, respectively. **d** Zebra plots were gated on CD4^+^ T cells, with the percentage of Th17 cells in CD4^+^ T cell population shown in the top right quadrants. Data were analyzed using paired *t*-test. (ns: not significant).

An Enhanced Th17 activation is usually accompanied with decreased Foxp3^+^ Tregs.^28^ However, staphylococcal superantigens have been shown to expand Foxp3^+^ Tregs in human PBMCs.^20,21^ We examined whether SAg-*Sau* stimulation affected Treg cell population in human NALT. Consistent with the superantigenic effects in human PBMCs, SAg-*Sau* stimulation led to expansion of the Foxp3^+^ Treg population, and this was significantly stronger compared to NonSAg-*Sau* and *Spn* (Fig. 2a, Supplementary Fig. 1a). Interestingly, IL-17A expression was increased markedly in the expanded Foxp3^+^ Tregs (Fig. 2a, Supplementary Fig. 1c), suggesting an unconventional Foxp3^+^ Treg population was induced in human NALT by SAg-*Sau* stimulation.

*Spn*-activated Tregs have been previously shown to be highly suppressive of T cell response.^29^ To determine whether SAg-*Sau*-expanded Tregs are functionally competent, the suppressive effect of Foxp3^+^ Tregs was then examined by assessing CD4^+^ T cell proliferative response in tonsillar MNCs with or without Foxp3^+^ Tregs. By depleting CD25^+^ cells, over 95% of Foxp3^+^ Tregs were removed from the MNCs (Supplementary Fig. 2a). Foxp3^+^ Treg-depleted MNCs and unfractionated MNCs were stained with CFSE and stimulated with SAg-*Sau* and *Spn*-TIGR4, respectively. CD4^+^ T cell proliferation was enhanced in Treg-depleted MNCs compared to unfractionated MNCs following *Spn*-TIGR4 stimulation, however SAg-*Sau*-activated CD4^+^ T cell proliferation was not affected by Treg removal (Fig. 2b). These results suggest an impaired suppression in NALT CD4^+^ T cell activation despite a highly expanded Foxp3^+^ T cell population, however this phenomenon appears to be specific to SAg-*Sau*.

Intriguingly, upon examining Th1 and Th17 responses, Treg depletion resulted in higher numbers of IFNγ^+^CD4^+^ Th1 cells but fewer IL-17A^+^CD4^+^ Th17 cells as compared to unfractionated MNCs upon SAg-*Sau* stimulation (Fig. 2c, d). Further examination revealed Treg depletion has resulted in the reduction of IL-17A^+^Foxp3^+^ T cells following stimulation. However, the proportion of IL-17A^+^Foxp3^−^ T cells were not affected by Treg removal (Supplementary Fig. 2b). Increased IFNγ (mean increase: 3830.2 pg/ml) and decreased IL-17A (mean decrease: 585.2 pg/ml) concentrations were also detected in the culture supernatant of Treg-depleted MNCs (*n* = 3, *p* < 0.05). Overall, the findings indicate that tonsillar Foxp3^+^ Tregs are able to suppress the Th1 activation but are unable to control the Th17 activation elicited by SAg-*Sau*.

To determine whether this observation was distinct to the nasopharynx immune microenvironment, we further examined the regulatory role of Foxp3^+^ Tregs in peripheral blood mononuclear cells (PBMCs). Consistent with the findings obtained from tonsillar MNCs, Treg depletion in PBMCs also enhanced Th1, but reduced Th17 response upon SAg-*Sau* stimulation (Supplementary Fig. 3). Thus, despite their critical role in maintaining immune tolerance, Foxp3^+^ Tregs were unable to inhibit SAg-*Sau*-activated Th17 responses in humans.

### SAg-*Sau* stimulation upregulates IL-10 but downregulates IL-35 expression in human tonsillar CD4^+^ T cells

**Fig. 3:**
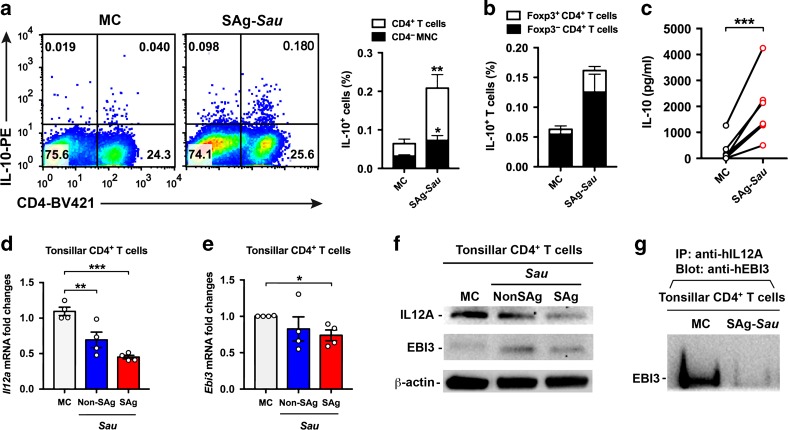
SAg-*Sau* stimulation upregulates IL-10 but downregulates IL-35 expression in human tonsillar CD4^+^ T cells. **a** IL-10 expression in tonsillar lymphocytes 48 h following SAg-*Sau* stimulation. Representative dot plots with numbers in top left and right quadrants indicate the percentage of IL-10^+^ CD4^−^ and IL-10^+^ CD4^+^ T cells within total lymphocyte population. Results from 8 individual samples were analyzed and summarized in the bar chat. **b** Bar chart showing the percentage of IL-10^+^ cells within Foxp3^+^ CD4^+^ T cell and Foxp3^−^ CD4^+^ T cell populations, respectively, *n* = 3. **c** IL-10 concentration in the culture supernatant of 48 h-cultured MNCs either unstimulated (MC) or stimulated with SAg-*Sau*. Samples were measured by ELISA in duplicate, *n* = 7. **d, e** Tonsillar MNCs were stimulated with SAg-*Sau* CCS for 24 h following which CD4^+^ T cell isolation was performed. mRNA was extracted from isolated CD4^+^ T cells for RT-qPCR. The fold change in *Il12a* (**d**) and *Ebi3* (**e**) mRNA expression compared to the media control (MC) are shown. Tests were performed in duplicate, *n* = 4. **f, g** Tonsillar MNCs were stimulated with SAg-*Sau* for 48 h and treated with brefeldin A for 4 h before harvesting cells for CD4^+^ T cell isolation. **f** Protein expression of IL-12A and EBI3 subunits in isolated CD4^+^ T cells. **g** CD4^+^ T cell lysate was immunoprecipitated with anti-IL-12A and blotted with anti-EBI3 to detect the expression of IL-35 heterodimer in CD4^+^ T cells. The blot image (**f, g**) are representative of 3 independent experiments. Data (**a, b, c, d, e**) were analyzed using paired *t*-test.

*Sau* infection has been shown to promote IL-10 production, but whether it alters IL-35 expression in CD4^+^ T cells is unknown.^24^ Expressions of IL-10 and IL-35 in tonsillar CD4^+^ T cells in response to SAg-*Sau* stimulation were examined. IL-10 expression in CD4^+^ T cells, primarily within Foxp3^−^ Tr1 cells, was significantly increased following SAg-*Sau* stimulation, and this was consistent with increased production of IL-10 in culture supernatants of stimulated MNCs as measured by ELISA (Fig. 3a–c). IL-35 is a heterodimeric cytokine consisting of IL12A and EBI3 subunits. Both subunits were detected by RT-qPCR and Western Blot in isolated CD4^+^ T cell with or without SAg-*Sau* stimulation and the existence of IL-35 heterodimer was confirmed using co-immunoprecipitation (Co-IP). The EBI3 subunit showed a slight downregulation at mRNA level. However, IL12A expression was substantially reduced at both mRNA and protein level, which resulted in downregulated production of IL-35 heterodimer in SAg-*Sau*-stimulated tonsillar CD4^+^ T cells (Fig. 3d–g). These results indicate SAg-*Sau* stimulation has opposite effect on IL-10 (enhanced) and IL-35 (reduced) production in human tonsillar CD4^+^ T cells.

### IL-10 suppresses SAg-*Sau*-activated Th1 response but not the Th17 response

**Fig. 4:**
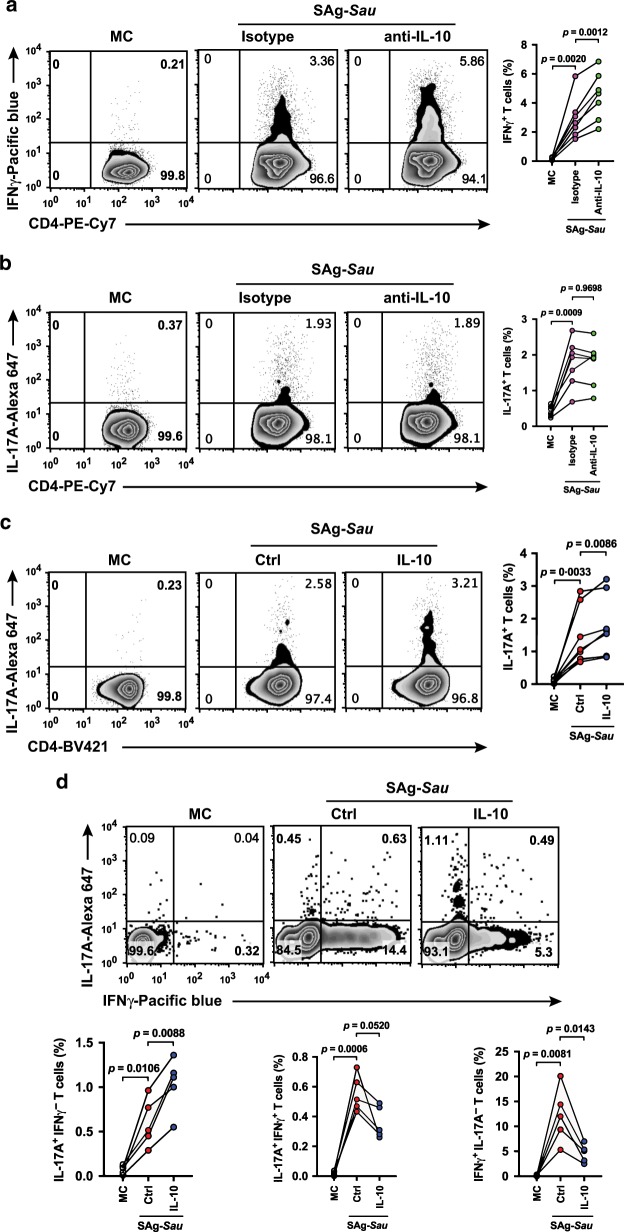
IL-10 suppresses SAg-*Sau*-activated Th1 response but not the Th17 response. **a, b** Analysis of IL-17A or IFNγ expressing CD4^+^ T cell responses in SAg-*Sau* stimulated tonsillar MNCs treated with IL-10 neutralizing antibody or isotype control. MC is unstimulated media control. Zebra plots were gated on CD4^+^ T cells and numbers in top right quadrants indicate the percentages of Th1 (**a**) or Th17 (**b**) within CD4^+^ T cell population. Data was analyzed using paired *t*-test with *p* values indicated, *n* = 7. **c**, **d** CD69^+^ cell depleted tonsillar MNCs were stimulated with SAg-*Sau* CCS in the presence or absence of recombinant IL-10 (10 ng/ml) for 48 h. Ctrl is the stimulation control. **c** Numbers in top right quadrants of the zebra plots indicate the percentage of Th17 in CD4^+^ T cells. Results of 8 individual samples were analyzed and summarized in symbol and line plot. **d** The percentage of IL-17A^+^IFNγ^-^, IL-17A^+^IFNγ^+^, and IL-17A^-^IFNγ^+^ cells within CD4^+^ T cell population is shown in top left, top right and bottom right quadrants of the zebra plots. Summarized data is displayed in symbol and line plot, *n* = 5.

The upregulated IL-10 expression in tonsillar MNCs following SAg-*Sau* stimulation may be a self-regulatory mechanism to suppress the highly inflammatory response that presides. By neutralizing IL-10 produced in the culture supernatant, the regulatory effects of IL-10 on both Th1 and Th17 activation were examined. Following IL-10 neutralization, the number of IFNγ-producing Th1 cells was increased in SAg-*Sau* stimulated tonsillar MNCs, however the proportion of IL-17A-producing Th17 cells was unchanged (Fig. 4a, b). This suggests that despite upregulated IL-10, Tr1 cells could not suppress SAg-*Sau*-activated Th17 response. IL-10 mediated regulation of Th1 and Th17 responses was further examined by adding recombinant IL-10 to MNCs stimulated with SAg-*Sau*. Again, addition of IL-10 suppressed Th1 activation (Supplementary Fig. 4), and in fact enhanced SAg-*Sau*-activated Th17 responses (Fig. 4c). The Th1 and Th17 cell populations were then divided into IL-17A^+^IFNγ^−^, IL-17A^+^IFNγ^+^, and IL-17A^−^IFNγ^+^ subpopulations to further define the opposing roles of IL-10 on regulation of IFNγ and IL-17A expression in SAg-*Sau*-activated CD4^+^ T cells. IL-17A^−^IFNγ^+^ cells were significantly reduced, and IL-17A^+^IFNγ^+^ cells were also decreased although this did not reach significance. However, a marked increase in IL-17A^+^IFNγ^−^ cell population was observed in IL-10-treated cells, providing further evidence that IL-10 could promote IL-17A expression in SAg-*Sau*-activated tonsillar CD4^+^ T cells (Fig. 4d).

### IL-35 suppresses over-reactive Th17 responses in SAg-*Sau* stimulated tonsillar MNCs

**Fig. 5:**
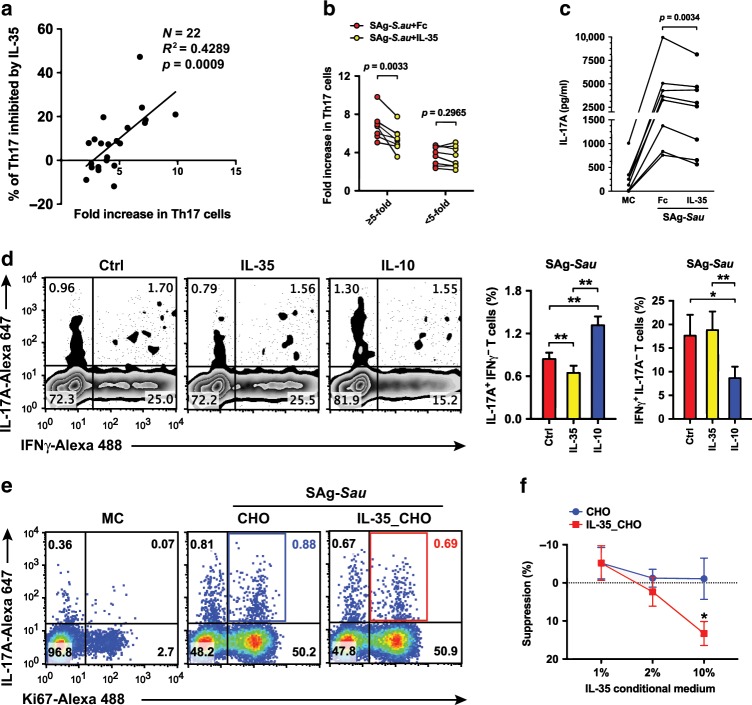
IL-35 suppresses over-reactive Th17 responses in SAg-*Sau* stimulated tonsillar MNCs. **a–c** Tonsillar MNCs were stimulated with SAg-*Sau* CCS for 48 h with 100 ng/ml of recombinant IL-35 or Fc control protein. **a** The association between fold increase in Th17 proportion and Th17 inhibition by IL-35. Each dot represents an individual sample, *n* = 22. Data was analyzed using linear regression. **b** The proportion of Th17 cells in total CD4^+^ T cells is shown as fold increase against media control (MC). The line plot shows the suppressive effect of IL-35 on Th17 responses in high responder group (≥5-fold) and low responder group (<5-fold), respectively, *n* = 8 per group. **c** IL-17A concentration in MNC culture supernatant of the high responders (*n* = 8) measured by ELISA, tests were performed in duplicate. **d** Inhibition of SAg-*Sau* activated Th17 and Th1 responses by IL-35 or IL-10 in tonsillar MNCs. Zebra plots were gated on CD4^+^ T cells and numbers in top left and bottom right quadrants indicate the percentage of IL-17A^+^ IFNγ^−^ and IL-17A^−^ IFNγ^+^ cells respectively in CD4^+^ T cell population. Data of 5 individual samples was analyzed using paired *t*-test and summarized in bar chats. **e**, **f** Tonsillar MNCs were stimulated with SAg-*Sau* (1 µg/ml) in the presence of conditioned medium from IL-35-transfected CHO cells (Clone 7) or control CHO cells at 1, 2, and 10% respectively. Five individual samples were tested and analyzed. **e** Representative dot plots were gated on CD4^+^ T cells and numbers in top right quadrants indicate the percentage of IL-17A^+^ Ki67^+^ cells (Proliferating Th17 cells) in CD4^+^ T cells. **f** Suppression (%) of Th17 proliferation in IL-35-transfected CHO cell medium-treated or control CHO cell medium-treated MNCs was calculated against stimulated MNCs without CHO cell medium, mean ± SEM is shown.

The degree of Th17 activation elicited by SAg-*Sau* in tonsillar MNCs varied from 2 to 10-fold among the individuals recruited in our study (Fig. 5a). To determine whether IL-35 downregulation in tonsillar CD4^+^ T cells by SAg-*Sau* was contributing to the highly activated Th17 responses, tonsillar MNCs were stimulated with SAg-*Sau* in the presence/absence of recombinant IL-35. Interestingly, the percentage of IL-35-mediated Th17 suppression was shown to be positively correlated with the level of Th17 activation (Fig. 5a). Tonsillar samples were then divided into high responders (≥ 5-fold increase) and low responders (<5-fold increase) based on the average fold increase in the proportion of Th17 cells within SAg-*Sau*-activated CD4^+^ T cell population. As shown in Fig. 5b, addition of IL-35 significantly reduced Th17 activation in the high responder group, whereas there was no effect on the low responders. Consistent with this, IL-17A production was also decreased in culture supernatants of high responders following IL-35 treatment (Fig. 5c). In contrast to IL-10, IL-35 suppressed the IL-17A^+^IFNγ^−^ cells without affecting the IL-17A^−^IFNγ^+^ CD4^+^ T cell population (Fig. 5d). These results suggest that IL-35 is able to suppress over-reactive Th17 responses while maintaining a low level of Th17 activation required for host defense in the human NALT.

The recombinant IL-35 used for the above experiments was an Fc fusion protein with linked EBI3 and IL-12A, which is widely used to study the biological function of IL-35.^25,30,31^ Although an Fc control protein was used at all times to exclude possible biological effects from the human IgG1 Fc protein region included in the recombinant protein and Fc treatment *per se* did not show any effect on SAg-*Sau*-activated T cell responses (data not shown), the way that protein subunits were fused together may also have impact on the biological activity of recombinant IL-35. To confirm that IL-35 mediated suppression of SAg-*Sau* activated Th17 response in tonsillar MNCs was not due to artificial IL-35-Fc fusion protein, a transfected CHO cell line producing native form of IL-35 was constructed by introducing IL-12A and EBI3 expressing plasmids into CHO cells, respectively. Both IL-12A and EBI3 proteins were expressed in 4 transfected CHO cell colonies selected (C7, C8, C14, and C15), and heterodimeric IL-12A/EBI3 protein secretion was confirmed by Co-IP Western Blot (Supplementary Fig. 5). The conditioned medium generated by C7 CHO cells, which produced the highest amount of IL-35 heterodimer, was used to examine the suppressive activity of native IL-35 on SAg-*Sau*-stimulated Th17 response. Tonsillar MNCs were cultured in medium containing C7 CHO cell or control CHO cell conditioned medium (either 1, 2, or 10%) and stimulated with SAg-*Sau*. MNCs stimulated without conditioned medium was used as stimulation control to calculate the suppression of Th17 responses by IL-35_CHO or control CHO cell conditioned medium. The native IL-35 showed inhibition of SAg-*Sau*-activated Th17 proliferation in a dose-dependent manner (Fig. 5e, f). Thus, it was confirmed that both IL-35-Fc and native form of IL-35 were functionally capable of regulating SAg-*Sau*-activated Th17 response in human tonsillar MNCs. Recombinant IL-35 with Fc fusion was used for subsequent experiments.

### IL-35 downregulates SAg-*Sau*-induced RORγt expression in human tonsillar CD4^+^ T cells

**Fig. 6:**
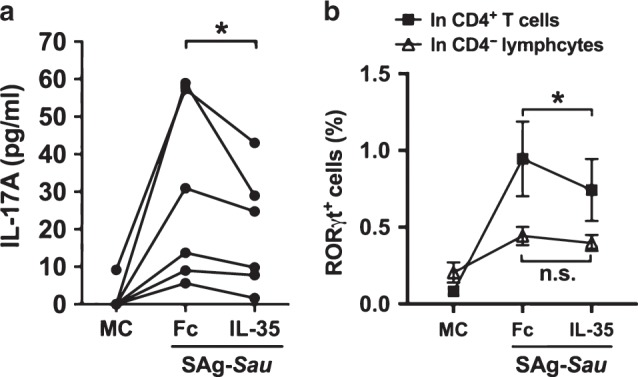
IL-35 downregulates SAg-*Sau* induced RORγt expression in human tonsillar CD4^+^ T cells. CD45RO^+^ cell depleted tonsillar MNCs were stimulated with SAg-*Sau* CCS (50 ng/ml) for 7 days in the presence of recombinant IL-1β (50 ng/ml), IL-21 (50 ng/ml), and TGFβ1 (2 ng/ml). 10 ng/ml of recombinant IL-35 or Fc control protein were added at day 0 and day 3. **a** IL-17A concentration in the cell culture supernatants as measured by ELISA, tests were performed in duplicate. Results of 6 individual samples were analyzed using paired *t*-test. **b** Line plot showing the percentage of RORγt^+^ CD4^−^ lymphocytes and RORγt^+^ CD4^+^ T cells in SAg-*Sau* stimulated tonsillar MNCs with or without IL-35. Data is shown in mean ± SEM, *n* = 4.

IL-35 has been reported to suppress Th17 cell differentiation.^32^ We therefore examined the effect of IL-35 on SAg-*Sau*-induced Th17 differentiation from naïve tonsillar CD4^+^ T cells. CD45RO^+^ cell depletion was performed to remove memory CD4^+^ T cells from tonsillar MNCs, and the depleted MNCs were then stimulated with SAg-*Sau* in the presence of Th17 polarizing cytokines (IL-1β, IL-21 and TGFβ1) for 7 days either with Fc control protein or IL-35. Fc treatment did not affect T cell responses in CD45RO^+^ cell depleted-MNCs following SAg-*Sau* stimulation. SAg-*Sau*-induced IL-17A production was significantly reduced by the addition of IL-35 (Fig. 6a). The expression of RORγt, the key transcription factor controlling differentiation of Th17 cells,^33^ was also downregulated by IL-35 in SAg-*Sau*-stimulated CD4^+^ T cells (Fig. 6b), suggesting that IL-35 could suppress SAg-*Sau*-induced Th17 differentiation via direct inhibition of RORγt expression.

### SAg-*Sau* colonization of mouse nasopharynx induces IL-17A expression and downregulates IL-12A in the cervical lymph node CD4^+^ T cells

**Fig. 7:**
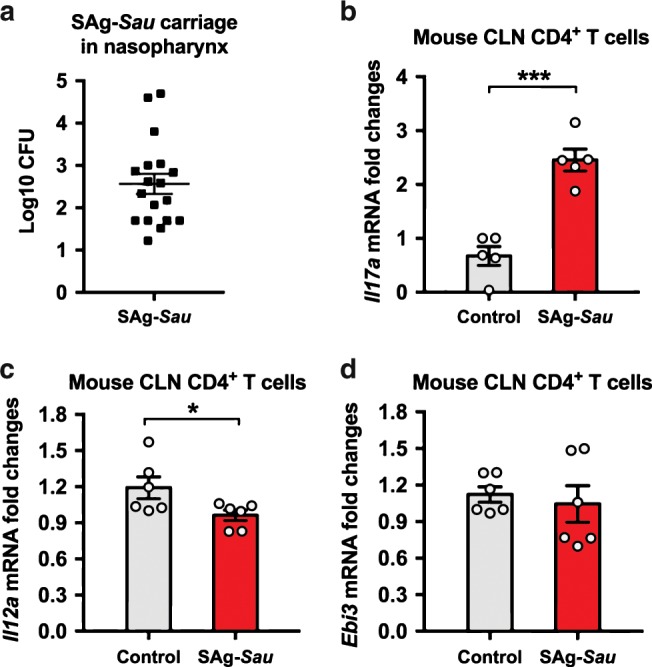
SAg-*Sau* colonization of the mouse nasopharynx induces IL-17A expression and downregulates IL-12A in the cervical lymph node CD4^+^ T cells. C57BL/6 mice were infected intranasally with 10^7^ CFU of SAg-*Sau* at day 0 and day 14, respectively. Nasopharynx and cervical lymph nodes (CLN) were harvested at 24 h post secondary infection. **a** Nasopharyngeal homogenates were plated on blood agar plates and colonies were counted after overnight culture. **b**–**d** CLN from 3 mice were pooled for lymphocyte isolation and CD4^+^ T cell separation. mRNA was extracted from separated CD4^+^ T cells for RT-qPCR. The mRNA expression of *Il17a* (**b**), *Il12a* (**c**), and *Ebi3* (**d**) are shown as fold changes against control mice. Results represent 2 independent experiments, 9 mice per group were used in each experiment. Data was analyzed using unpaired *t*-test and displayed as mean ± SEM.

As we had shown a highly activated Th17 response in human tonsillar MNCs driven by SAg-*Sau*, which was associated with downregulated IL-35 expression in the CD4^+^ T cells in vitro, a mouse nasal colonization model was used to further examine the in vivo activation of NALT CD4^+^ T cells. C57BL/6 mice were intranasally infected with SAg-*Sau* and Th17 response in cervical lymph nodes (CLN) was assessed after secondary infection. All mice infected with SAg-*Sau* carried the bacterium at 24 h post-secondary infection (Fig. 7a). Consistent with in vitro Th17 activation by SAg-*Sau* in human tonsillar MNCs, mice infected with SAg-*Sau* also had significantly higher *Il17a* mRNA expression in their CLN CD4^+^ T cells compared to their naïve counterparts (Fig. 7b). The mRNA expression of *Il12a* and *Ebi3* were also determined. Although less significant than in human tonsillar CD4^+^ T cells, the expression of *Il12a* in mouse CLN CD4^+^ T cells was also reduced by SAg-*Sau* colonization, while *Ebi3* expression remained unchanged (Fig. 7c, d). In humans, IL-35 is produced primarily by effector CD4^+^ T cells, however in mice, Foxp3^+^ Tregs constitutively express IL-35 and are the primary source of this cytokine.^34,35^ SAg-*Sau* stimulation led to a more significant decrease in IL-35 expression in human CD4^+^ T cells, suggesting that it may primarily downregulate IL-35 expression in effector CD4^+^ T cells. Despite this, both ex vivo human cell culture and in vivo mouse carriage models show that SAg-*Sau* colonization downregulates IL-35 expression in CD4^+^ T cells, which could contribute to an enhanced Th17 response.

## Discussion

The balance between regulatory and pro-inflammatory host T cell responses to SAg-*Sau* infection is likely to be critical in preventing excessive inflammation (e.g., toxic shock) and autoimmune conditions. Our study highlights a critical cellular regulatory mechanism on Th17 activation in human nasopharynx mucosal tissue, whereby IL-35 is identified as a key suppressive cytokine in controlling SAg-*Sau*-driven inflammatory Th17 responses. Although SAg-*Sau* induced a marked Treg expansion and enhanced IL-10 production (Figs. 2a and 3a-c), they failed to suppress SAg-*Sau*-activated Th17 responses. By contrast, IL-35 expression in tonsillar CD4^+^ T cells was markedly downregulated by SAg-*Sau* which was associated with an enhanced Th17 activation, and introduction of recombinant IL-35 suppressed the Th17 response. These findings suggest IL-35, but not Foxp3^+^ Treg or IL-10, is critical in suppressing SAg-*Sau*-activated inflammatory Th17 response in human NALT.

We demonstrate SAg-*Sau* activated a potent Th17 response in NALT, which is consistent with previous findings showing *Sau* superantigens activate Th17 cells.^11,36^ Nevertheless, non-SAg-*Sau* strains were also shown to activate a Th17 response, although in general significantly less than SAg-*Sau* strains. It is possible that some non-superantigenic *Sau* properties could also contribute to Th17 activation.

Tregs in human PBMCs have been shown to suppress proliferation of CD25^−^CD4^+^ T cells activated by anti-CD3 but were unable to suppress staphylococcal superantigen-induced T cell proliferation.^21^ Consistent with this finding, we show NALT-derived Tregs failed to suppress SAg-*Sau*-activated proliferative response. Interestingly, further examination revealed that Tregs effectively suppressed IFNγ-producing Th1 cells but were unable to control the activation of IL-17A-producing Th17 cells. SAg-*Sau*-expanded Tregs exhibited markedly decreased expression of CD39 (Supplementary Fig. 6), which is an ectonucleotidase converting extracellular ATP to suppressive adenosine with the help of CD73.^37^ Both CD39^+^ and CD39^−^ Tregs were shown to inhibit Th1 response but only CD39^+^ Tregs were capable of suppressing Th17 response in human PBMCs.^38^ The lack of CD39^+^ Tregs following SAg-*Sau* stimulation may help explain the Treg-mediated differential suppression of Th1 and Th17 responses shown in our study.

In addition to expanded Foxp3^+^ Tregs, SAg-*Sau* also upregulated IL-10 expression mostly by Foxp3^−^ CD4^+^ T cells (Tr1) in tonsillar MNCs (Fig. 3a, b). An intriguing finding in our study was that SAg-*Sau*-activated Th17 cells were resistant to IL-10-mediated suppression, while Th1 cells remained sensitive. IL-10/IL-10R signaling in Tregs and Th17 cells has been reported to mediate suppression of inflammatory Th17 activation.^39,40^ SAg-*Sau* stimulation may impair the IL-10 signaling in activated Th17 cells making them unresponsive to IL-10-mediated suppression.

More intriguingly, we demonstrate that the addition of recombinant IL-10 further promoted IL-17A expression in SAg-*Sau*-stimulated tonsillar CD4^+^ T cells. It is plausible that plasticity of Th17 leads to the generation of an IL-17A^+^IFNγ^+^ (Th17/Th1) cell subset, this may be associated with an enhanced STAT1 activation in the cells which limits STAT3 activity and its downstream IL-17 expression. If STAT1 signaling keeps pushing over and perturbs the delicate balance of STAT1 and STAT3 in the Th17/Th1 cells, these cells may lose expression of Th17-related genes and become ex-Th17 cells.^41^ However, inhibiting STAT1 activation could remove its suppression over STAT3 and thereby revive Th17 effector phenotype.^42^ Hence, in SAg-*Sau*-stimulated MNC, exogenous IL-10 may promote IL-17A expression in Th17/Th1 and ex-Th17 subsets through downregulating IFNγ and STAT1 activation, thus leading to an enhanced IL-17A^+^IFNγ^-^, but a reduced IL-17A^+^IFNγ^+^ cell subset.

Neither Foxp3^+^ Tregs nor IL-10 was able to control SAg-*Sau*-activated potent Th17 responses. This led us to examine the role of another regulatory cytokine, IL-35, which has been shown to inhibit autoimmunity by suppressing inflammatory Th17 activation in murine studies.^25,35,43,44^ We show IL-35 heterodimer was expressed in human tonsillar CD4^+^ T cell without stimulation and its expression was significantly downregulated upon SAg-*Sau* stimulation. This raised the possibility that loss of IL-35 expression may contribute to the strong Th17 response activated by SAg-*Sau*. Indeed, addition of IL-35 suppressed SAg-*Sau*-activated Th17 responses which positively correlated with the level of Th17 activation (Fig. 5a). Depending on the closeness between IL-35-producing cells and Th17 cells, the biological concentration of IL-35 in physiological condition to inhibit Th17 cells could be hard to determine. The concentration of recombinant IL-35 we used to treat cells was carefully titrated based on published IL-35 studies.^25,43^ The finding that recombinant IL-35 selectively suppressed those with high Th17 responses following SAg-*Sau* stimulation suggests IL-35 may regulate physiological Th17 activation and potentially pathogenic Th17 activation through different signaling pathways.

IL-10-dependent and Treg-dependent mechanisms have been reported to play a role in IL-35-mediated Th17 suppression.^45,46^ We have shown that IL-10-mediated and Treg-mediated suppression on effector Th17 cells were impaired by SAg-*Sau* stimulation, which may explain why IL-35 did not suppress SAg-*Sau*-activated low Th17 responses. IL-35 could signal through a unique IL-12Rβ2:gp130 heterodimer or via homodimers to suppress effector T cell activation, but only via the heterodimeric receptor can it induce expression of IL-35.^47^ It is possible that IL-35-mediated Th17 suppression require high expression of IL-12Rβ2:gp130 heterodimer and through induction of IL-35 in targeted Th17 cells. Similar to IL-10 expression in Th17 cells, which directs the cell towards a regulatory phenotype,^48^ IL-35 expression in pathogenic Th17 cells may also elicit an anti-inflammatory gene expression profile to reverse the post-activation fate of pathogenicity.

In conclusion, we identify IL-35, but not IL-10, as a critical cytokine in controlling SAg-*Sau*-activated Th17 responses in human nasopharynx. This finding may also apply to other mucosal and systemic compartments where SAg-*Sau* or superantigens could cause Th17-mediated inflammation. The results may have therapeutic implications for IL-35 in future management of SAg-*Sau*-induced inflammatory disease (e.g., toxic shock) or autoimmune conditions.

## Methods

### Patients and samples

The adenotonsillar tissues were obtained from immunocompetent patients undergoing adenoidectomy and/or tonsillectomy due to upper-airway obstructions at Alder Hey children’s Hospital, and Royal Liverpool and Broadgreen University Hospitals. Patients with known immunodeficiency or who had been prescribed antibiotics within 3 weeks before surgery were excluded. Nasopharyngeal swabs were taken on the day of operation and stored in STGG medium at −80 °C before bacterial culture as described previously.^49^ The study was approved by the National Research Ethics Committee and written consent was obtained in all cases. In total, adenotonsillar tissue samples from 65 patients were used in this study.

### Mouse nasal colonization model

Female C57BL/6 mice aged 6–8 weeks were purchased from Charles River, UK. Mice were maintained in individually ventilated cages at 22 ± 1 °C and 65% humidity with a 12 h light-dark cycle. Mice were infected intranasally with 10^7^ colony-forming unit (CFU) of SAg-*Sau* in 10 μl PBS or PBS only for control group mice. A second dose of SAg-*Sau* was given at day 14 post primary infection. Mice were then culled at 24 h post-secondary infection to assess the nasopharyngeal bacteria load and CD4^+^ T cell responses in the cervical lymph nodes (CLNs). All experimental protocols were approved and performed in accordance with the regulations of the Home Office Scientific Procedures Act (1986), project licence P86De83DA and the University of Liverpool Ethical and Animal Welfare Committee.

The nasopharyngeal tissue was removed and homogenized in 3 ml of sterile PBS before plating out on blood agar for assessment of tissue CFU. Lymphocytes were isolated from the dissected CLN, followed by CD4^+^ T cell separation using positive selection microbeads (Miltenyi). RT-qPCR was performed with isolated CLN CD4^+^ T cells (purity > 96%) to detect gene expression.

### Preparation of bacterial culture supernatant

Pneumococcal strains used in this study were standard encapsulated serotype 2 (D39) strain and virulent serotype 4 (TIGR4) strain. For *S. aureus*, we included Superantigenic strain FRI913 (positive for tsst, sea, sec, and see), Non-Superantigenic strain HIP07930 (negative for PVL, tsst, sea, seb, sec, sed and see) (BEI Resources) and 3 carriage strains (*Sau*-C1, *Sau*-C2, and *Sau*-C3) isolated from the nasopharyngeal swabs of subjects from this study. Strains *of M. catarrhalis* and Coagulase-Negative *Staphylococcus* (CNS) (CNS-C4 and CNS-C5) were also carriage isolates from the study subjects. Bacteria were cultured in Todd-Hewitt broth (Oxoid, Basingstoke, UK) supplemented with 0.5% yeast extract at 37 °C until the OD_620_ reaches 0.5. Bacterial culture supernatant was prepared and concentrated as described previously.^49^ The protein concentration of the concentrated culture supernatant (CCS) was measured using Pierce^TM^ BCA Protein Assay Kit (Thermo Fisher Scientific). The RIDASCREEN^@^ SET Total Kit (r-biopharm, Germany) was used to detect total staphylococcal enterotoxin A-E level in the culture supernatant of 3 carriage strains.

### Human tonsillar mononuclear cell isolation, culture, and stimulation

Tonsillar mononuclear cells (MNCs) were isolated using methods described previously.^49^ Briefly, palatine tonsil was minced in Hank’s buffer with a sterile scalpel before filtering through a 70 nm cell strainer. MNCs were isolated by Ficoll density gradient centrifugation (GE Healthcare). Cells were washed and suspended in RPMI1640 (Thermo Fisher Scientific) supplemented with 10% fetal bovine serum (Sigma Aldrich), 2 mmol/L glutamine 100 U/ml penicillin and 100 μg/ml streptomyicin (Thermo Fisher Scientific). In some experiments, CD69^+^, CD45RO^+^ cells or CD25^+^ cells were depleted from the MNCs by magnetic cell sorting (Miltenyi Biotec, Surrey, UK) according to the manufacturer’s instructions. Purity of isolated CD69^−^ MNCs (>95%), CD45RO^−^ MNCs (>99%) and Treg-depleted MNCs (>95%) following CD25^+^ cell depletion was confirmed by flow cytometry. The tonsil MNCs were cultured at 4 × 10^6^ cells/ml in 48-well plate and stimulated with 1 μg/ml bacterial CCS for 48 h to examine memory T cell responses. In IL-10 neutralization experiment, cells were treated with 1 μg/ml of LEAF purified anti-human IL-10 or rat IgG1κ isotype ctrl antibody (BioLegend). In some experiments, 100 ng/ml of recombinant IL-10 and IL-35 were added to the cell culture, respectively. CD45RO^+^ cell-depleted MNCs were cultured in the presence of 2 ng/ml of TGF-β1, 50 ng/ml of IL-1β, and 50 ng/ml of IL-21 and stimulated with 50 ng/ml bacterial CCS for 7days to induce Th17 cell differentiation, with recombinant IL-35 or Fc control protein being added at day 0 and day 3. Recombinant IL-10 was purchased from PeproTech and other recombinant proteins were from R&D systems.

### Intracellular cytokine staining

Tonsillar MNCs were treated with Brefeldin A (eBiosciences) for 4 h before harvesting cells in order to block cytokine secretion. MNCs were stained with fluorescence labeled anti-human CD4, followed by fixation and permeabilization and staining intracellularly with fluorescence labeled anti-human Foxp3, IL-17A, IFNγ, IL-10, RORγt, and Ki67 in different combinations. Stained cells were acquired on BD Celesta or BD Canto II and data was analyzed by Flowjo. Anti-human CD4-PECy7, IL-17A-Alexa Fluor 647, Foxp3-Alexa Fluor 488, IL-10-PE, and RORγt-PE FACS antibodies were from BD Biosciences. Other antibodies were purchased from BioLegend.

### Quantitative reverse transcription polymerase chain reaction (RT-qPCR)

Human tonsillar CD4^+^ T cells were isolated from 24 h stimulated tonsillar MNCs by magnetic cell sorting with a purity of > 99%. 1 × 10^6^ isolated CD4^+^ T cells were used for total RNA extraction with RNeasy kit (Qiagen) according to the manufacturer’s instructions. cDNA was synthesized using Promega kit and qPCR was performed using SYBR^®^ Green JumpStart^TM^ Taq ReadyMix^TM^ (Sigma) on Bio-Rad CFX96^TM^. Gene expression in mouse CLN CD4^+^ T cells was also examined as described above. All qPCR reactions were performed in duplicate.

Primers used for qPCR are displayed in Table 1. mRNA expression levels were normalized to the levels of human or mouse *βactin* housekeeping gene and calculated using 2^-ddCT^ formula.

**Table 1:** qPCR Primers (synthesized by Sigma Aldrich).

Gene	Primers		T_m_ (°C)	Product size (bp)
Human *il12a* NM_000882	Forward	5’-TGCCTTCACCACTCCCAAAACCTGCTGA-3’	70.0	154
	Reverse	5’-ATGGTAAACAGGCCTCCACTGTGCTGGT-3’	69.6	
Human *ebi3* NM_005755.2	Forward	5’-AAACTCCACCAGCCCCGTGTCCTTCATT-3’	69.9	161
	Reverse	5’-CGGTGACATTGAGCACGTAGGGAGCCAT-3’	70.1	
Human *βactin* NM_001101.5	Forward	5’-GCTCACCATGGATGATGATATCGCCGC-3’	67.7	199
	Reverse	5’-GATGCCTCTCTTGCTCTGGGCCTCGTC-3’	70.2	
Mouse *il17a* NM_010552.3	Forward	5’-CCAGCTGATCAGGACGCGCAAACATGAG-3’	69.7	115
	Reverse	5’-TGAGGGATGATCGCTGCTGCCTTCACTG-3’	70.1	
Mouse *il12a* NM_001159424.2	Forward	5’-TTCCAGGCCATCAACGCAGCACTTCAGA-3’	70.2	196
	Reverse	5’-TGAAGGCGTGAAGCAGGATGCAGAGCTT-3’	70.3	
Mouse *ebi3* NM_015766.2	Forward	5’-GCCTCCTAGCCTTTGTGGCTGAGCGAAT-3’	70.3	133
	Reverse	5’-GAGAAGATGTCCGGGAAGGGCCAGGAAG-3’	69.8	
Mouse *βactin* NM_007393.5	Forward	5’-TTCTTTGCAGCTCCTTCGTTGCCGGTC-3’	69.5	199
	Reverse	5’-CCTTCTGACCCATTCCCACCATCACACC-3’	68.6	

### Expression of recombinant human IL-35 in its native form in CHO cell

Expression plasmids containing genes encoding human EBI3 (pRP-Neo-hEBI3) and IL-12A (pRP-Puro-hIL12A) were constructed by VectorBuilder. Plasmids were introduced into CHO cells using Lipofectamine3000 (Invitrogen) cell transfection kit. Successful transfectants were selected and expanded via G418 and puromycin double selection. Co-expression of human EBI3 and IL-12A in transfected clones was confirmed by Western blotting. The heterodimeric EBI3/IL-12A (IL-35) secretion was detected by co-immunoprecipitation and Western blotting. IL-35-expressing CHO cell clones and control CHO cells were cultured in a comparable cell density. The cell culture medium collected at 48 h was used as conditioned medium to test the biological function of native IL-35 in this study.

### Immunoprecipitation and Western Blotting

Tonsillar MNCs were stimulated with *Sau* CCS for 48 h with the addition of Brefeldin A for the last 4 h followed by isolation of CD4^+^ T cells. For immunoprecipitation, equal number (1 × 10^7^) of isolated cells were lysed in 1 ml of RIPA buffer without SDS (Sigma). The cell lysis was sonicated and centrifuged to remove cell debris and DNA fragments before incubating with 1.5 μg/ml anti-human IL-12A mouse monoclonal antibody (mAb) (R&D, Clone 27537) at 4 °C for 1 h. 50 μl of buffer equilibrated Sheep anti-Mouse IgG conjugated Dynabeads M-280 (Life Technology) were added to the 1 ml cell lysis and incubated overnight at 4 °C to capture the target protein. Dynabeads were washed and heated at 100 °C for 10 min to elute antibody captured protein in reducing sample buffer. Protein samples were analyzed by standard Western Blotting using 1 μg/ml anti-human EBI3 (Biolegend, Clone A15058A). For the detection of IL-12A and EBI3 separately in stimulated CD4^+^ T cells or constructed CHO cell clones, standard Western Blotting was performed using above mentioned anti-IL-12A and anti-human EBI3 mAbs, respectively.

### Measurement of cytokine production

Tonsillar cell culture supernatant was collected following cell culture with or without stimulation. IL-17A and IL-10 concentrations in the culture supernatant were measured by ELISA kits (eBioscences) following the manufacturer’s instructions. All samples were tested in duplicate.

### Statistical analysis

Data distribution were tested using D'Agostino-Pearson or Shapiro-Wilk normality tests and results were then analyzed accordingly with paired *t*-test (two-tailed) or unpaired *t*-test (two-tailed). Data that is not normally distributed was log-transformed before performing parametric *t*-tests or analyzed using Wilcoxon matched-pairs signed rank test (two-tailed). The association between fold-increase in Th17 response and Th17 inhibition by IL-35 was analyzed using linear regression. All data was analyzed in Prism 7 software. Data displayed is mean ± SEM for column bar graphs and line plots. For data shown as box and whiskers, median (center line), upper and lower quartile (box limits) and minimum to maximum range (whiskers) are displayed. Asterisks denote *p*-value (*p *<* 0.05, **p < 0.01, ***p < 0.001, ****p *<* 0.0001). *p*-value < 0.05 were considered significant. n.s. means not significant.

## Supplementary information

Supplementary Information
