# Supplementary material for: IL-35 is critical in suppressing superantigenic Staphylococcus aureus-driven inflammatory Th17 responses in human nasopharynx-associated lymphoid tissue
Source: Mucosal Immunol. 2020 Jan 2;13(3):460–70. doi: 10.1038/s41385-019-0246-1 (PMC7181393; doi:10.1038/s41385-019-0246-1)
Supplement: Supplementary file 1 — Supplementary Information [file 41385_2019_246_MOESM1_ESM.docx]

**Supplementary information**


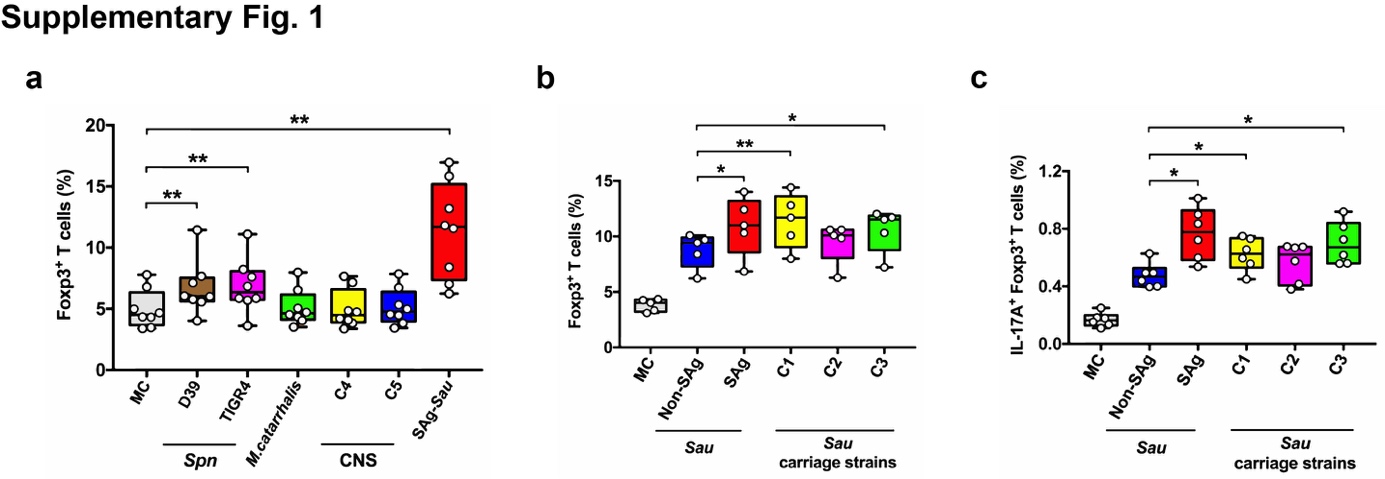


**Supplementary Fig. 1. SAg-*Sau* stimulation expands Foxp3^+^ Treg population and induces IL-17A^+^Foxp3^+^ CD4^+^ T cells in tonsillar MNCs.** Analysis of Treg expansion (**a, b**) and IL-17A-expressing Tregs (**c**) in isolated human tonsillar MNCs at 48hrs following bacterial CCS (1µg/ml) stimulation. **a)** Tonsillar MNCs were stimulated with CCS produced from *Spn*, *M. catarrhalis*, coagulase-negative staphylococcus (CNS, C4 and C5) and SAg-*Sau*, and the proportion of Tregs was analysed. Proportion of Tregs (**b**) and IL-17A-expressing Tregs (**c**) in CD4^+^ T cell population activated by NonSAg-*Sau*, SAg-*Sau* and *Sau* carriage strains (C1, C2 and C3). Results represent 8 (**a**), 5 (**b**) and 6 (**c**) independent experiments. Data displayed is median (center line), upper and lower quartile (box limits) and minimum to maximum range (whiskers). (^*^*p* <0.05, ^**^*p* <0.01)

**
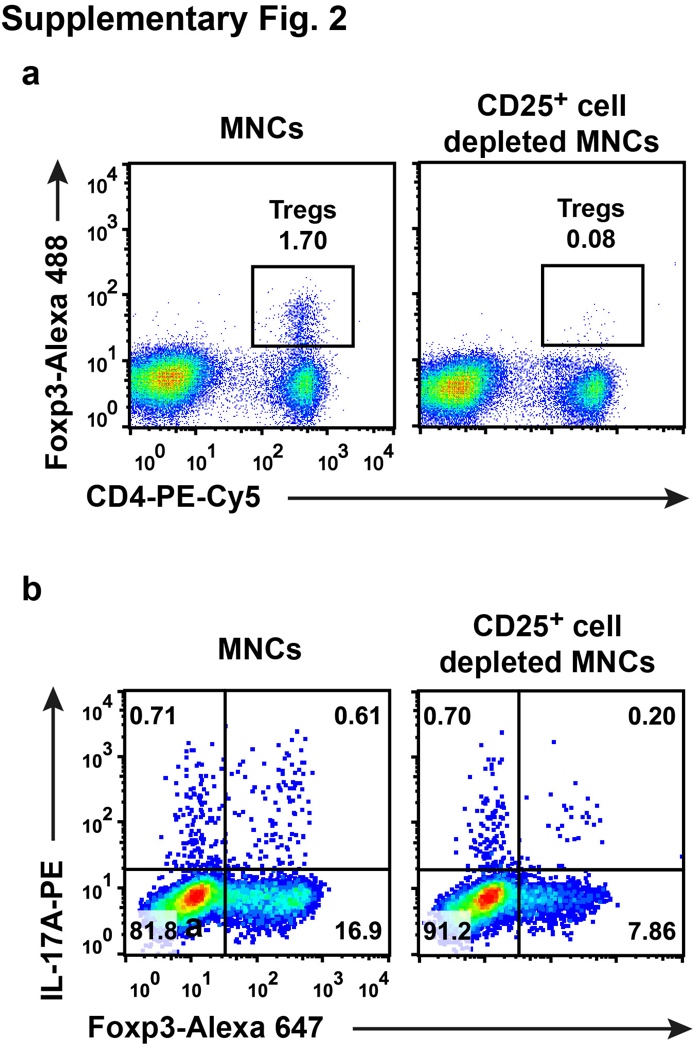
**

**Supplementary Fig. 2. CD25^+^ cell depletion removes Foxp3^+^ Tregs in tonsillar MNCs. a)** Foxp3^+^ CD4^+^ cells (Tregs) were gated out in the rectangular boxes with numbers on top indicating the percentage of Tregs in lymphocytes before and after CD25^+^ cell depletion. **b)** Unfractionated and CD25^+^ cell-depleted MNCs were stimulated with 1µg/ml of SAg-*Sau* CCS for 48hrs and activation of IL-17^+^ T cells was examined. CD4^+^ T cells were gated out in the representative dot plots and numbers in top right and left quadrants indicating percentages of IL-17A^+^ cells within Foxp3^+^ and Foxp3^-^ T cells respectively. Results are representative of 3 individual samples.


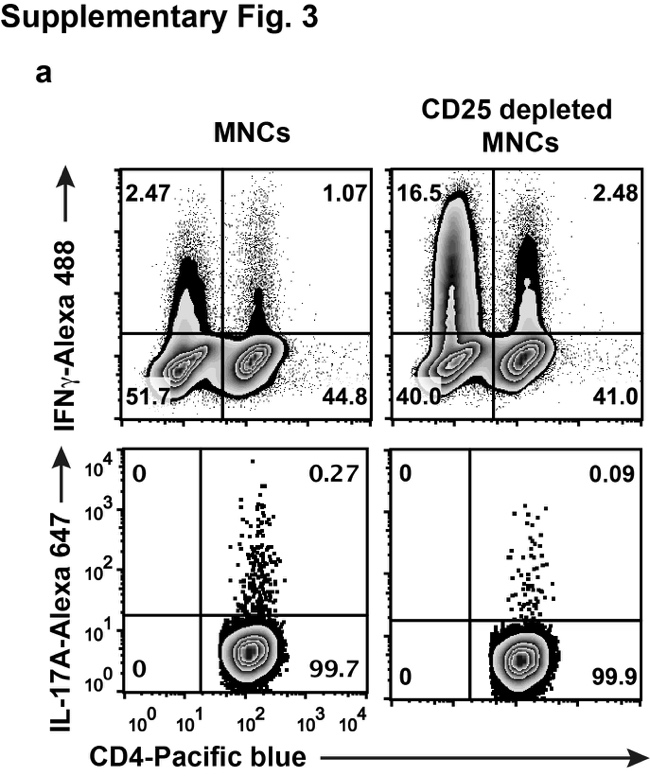


**Supplementary Fig. 3. Foxp3^+^ Tregs suppresses the Th1 but not Th17 responses in PBMCs activated by SAg-*Sau*.** IL-17A and IFNγ expression in unfractionated PBMCs or CD25^+^ cell depleted PBMCs stimulated with SAg-*Sau* CCS (1µg/ml) at 48hrs. **a)** Zebra plots were gated on lymphocytes for IFNγ expression. Numbers in top left and right quadrants indicate the percentage of IFNγ^+^ CD4^-^ lymphocytes and IFNγ^+^ CD4^+^ T cells (Th1) respectively. For the expression of IL-17A, zebra plots were gated on CD4^+^ T cells and the percentage of Th17 cells within CD4^+^ T cell population was indicated in the top right quadrants.


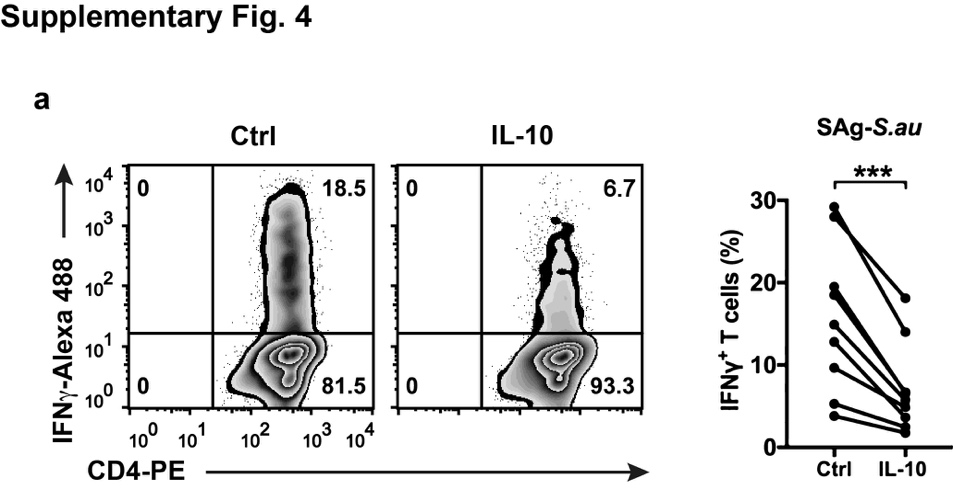


**Supplementary Fig. 4. IL-10 suppresses SAg-*Sau*-activated Th1 responses.** Zebra plots were gated on CD4^+^ T cells and numbers in top right quadrants indicate the percentage of Th1 cell within CD4^+^ T cell population. Ctrl is the stimulation control without IL-10 treatment. Results represent 8 independent experiments and were analysed using paired *t*-test (^***^*p* <0.001).


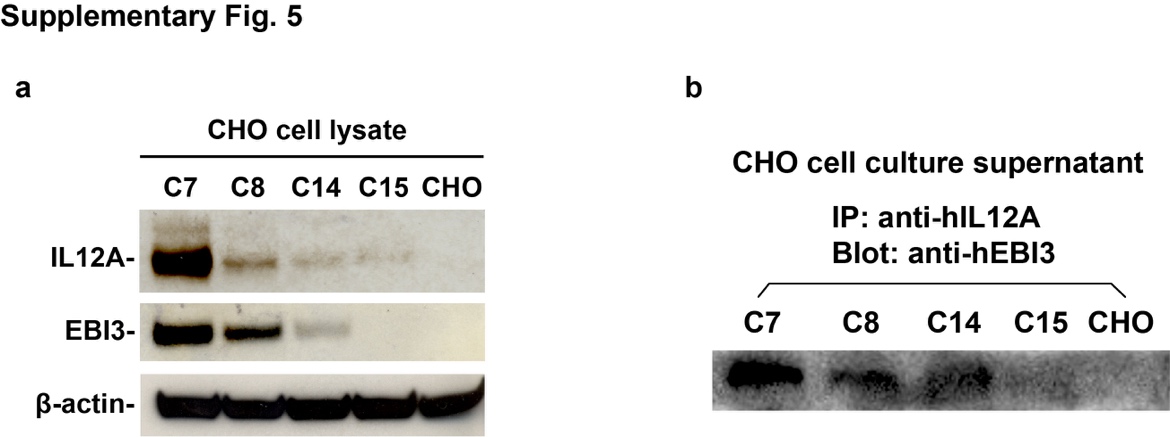


**Supplementary Fig. 5. Expression and secretion of native IL-35 by transfected CHO cells.** Control CHO and IL-35 expressing CHO cells (Clone 7, 8, 14, 15) were cultured for 48hrs. **a)** Protein expression of IL-12A and EBI3 in cell lysates. **b)** Production of IL-35 heterodimer in cell culture supernatant as detected by co-immunoprecipitation.


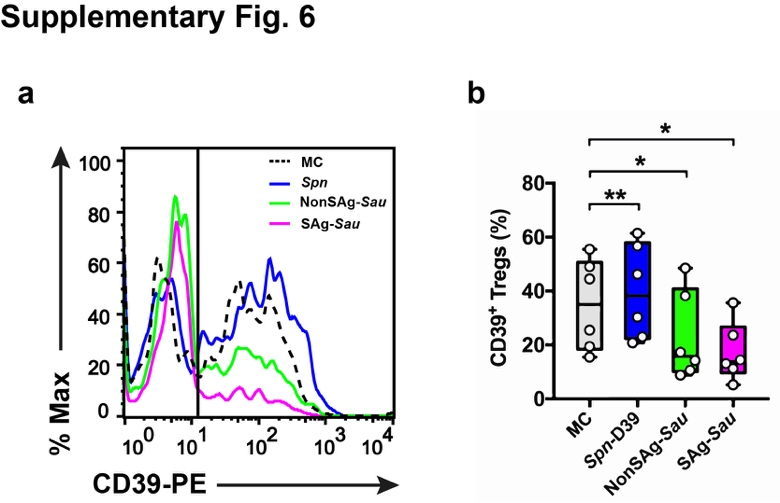


**Supplementary Fig. 6. SAg-*Sau* stimulation downregulates cell surface expression of CD39 on Foxp3^+^ Tregs.** Tonsillar MNCs were stimulated with 1µg/ml of *Spn*, NonSAg-*Sau* and SAg-*Sau* CCS respectively for 48hrs. Expression of CD39 was detected by cell surface staining and compare to media control (MC). **a)** Histogram plots were gated on Foxp3^+^ CD4^+^ cells, and the percentage of CD39^+^ cells within Tregs were analysed in (**b**). Results represent 6 independent experiments. Data displayed is median (center line), upper and lower quartile (box limits) and minimum to maximum range (whiskers). (^*^*p* <0.05, ^**^*p* <0.01)

**
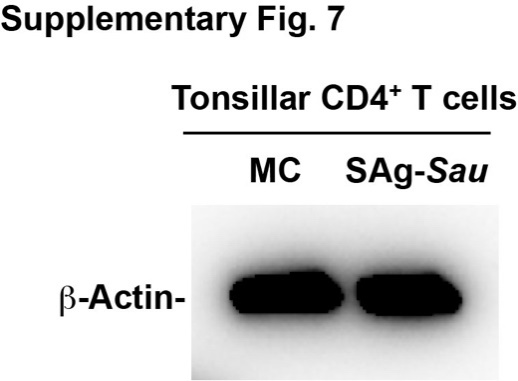
**

**Supplementary Fig. 7. β-actin expression in CD4^+^ T cell lysates.** β-actin was detected by Western blot for the CD4^+^ T cell lysates prepared for IL-35 immunoprecipitation assay.
